# The Neutrophil‐to‐Lymphocyte Ratio Is an Independent Inflammatory Biomarker for Adverse Events in Patients With Atrial Fibrillation: Insights From the Murcia AF Project II (MAFP‐II) Cohort Study

**DOI:** 10.1002/clc.70102

**Published:** 2025-02-22

**Authors:** Eva Soler‐Espejo, Francisco Marín, Raquel López‐Gálvez, María Pilar Ramos‐Bratos, María Sánchez‐Villalobos, María Asunción Esteve‐Pastor, Gregory Y. H. Lip, José Miguel Rivera‐Caravaca, Vanessa Roldán

**Affiliations:** ^1^ Department of Hematology Hospital Clínico Universitario Virgen de la Arrixaca, University of Murcia, Instituto Murciano de Investigación Biosanitaria (IMIB‐Arrixaca) Murcia Spain; ^2^ Department of Cardiology Hospital Clínico Universitario Virgen de la Arrixaca, University of Murcia, Instituto Murciano de Investigación Biosanitaria (IMIB‐Arrixaca), CIBERCV Murcia Spain; ^3^ Liverpool Centre of Cardiovascular Science at University of Liverpool, Liverpool John Moores University and Liverpool Heart and Chest Hospital Liverpool UK; ^4^ Department of Clinical Medicine Aalborg University Aalborg Denmark; ^5^ Faculty of Nursing University of Murcia Murcia Spain

**Keywords:** atrial fibrillation, cardiovascular death, major bleeding, mortality, neutrophil‐to‐lymphocyte ratio, stroke

## Abstract

**Background:**

Systemic inflammation plays a central role in atrial fibrillation (AF). The neutrophil‐to‐lymphocyte ratio (NLR) is a simple hematological index that has been shown to be associated with prognosis in different pathologies.

**Hypothesis:**

The NLR is associated with an increased risk of adverse events in patients with AF.

**Methods:**

We included a prospective cohort of AF patients who started vitamin K antagonists (VKAs) therapy between July 2016 and June 2018. NLR was assessed at baseline and classified into three categories: low (< 3), moderate (3–5), and high (> 5). During a 2‐year follow‐up period, all cardiovascular deaths, all‐cause deaths, and net clinical outcomes (NCO; either ischemic stroke/transient ischemic attack, major bleeding or all‐cause death), were recorded.

**Results:**

A total of 1050 patients were included (51.4% women; median age 77 years). NLR was available in 936 patients: 507 (54.2%) had low NLR (< 3), 239 (25.5%) had moderate NLR (3–5), and 190 (20.3%) had high NLR (> 5). The primary endpoint was significantly increased in the high NLR category (*p* = 0.002 for cardiovascular death; *p* < 0.001 for all‐cause mortality, and *p* < 0.001 for NCO), with higher IRRs (all *p* < 0.001). Multivariate Cox regression analyses showed that high NLR was independently associated with an increased risk of cardiovascular death (aHR: 2.02; 95% CI: 1.04–3.92), all‐cause mortality (aHR: 2.51; 95% CI: 1.58–3.97), and NCO (aHR: 1.99; 95% CI: 1.37–2.87), compared to low NLR.

**Conclusions:**

In this prospective AF cohort receiving VKAs, elevated NLR was significantly associated with an increased risk of adverse clinical outcomes. NLR has independent prognostic value beyond other classical risk factors.

## Summary

- Atrial fibrillation (AF) is associated to chronic inflammation, which increases the risk of thrombosis, and the neutrophil‐to‐lymphocyte ratio (NLR) has emerged as a potential biomarker for predicting adverse events in different diseases.
- Our study aimed to investigate the role of NLR as a predictive inflammatory biomarker for adverse events in a contemporary real‐world prospective cohort of AF patients.
- Among our cohort of AF patients, those in the high NLR category (> 5) presented a higher incidence rate ratio for cardiovascular death, all‐cause mortality, and net clinical outcomes (NCO).
- Higher NLR was independently linked to increased risks of cardiovascular death (aHR: 2.02), all‐cause mortality (aHR: 2.51), and NCO (aHR: 1.99), with a linear relationship between the NLR and the risk of these adverse events (*p*‐values for nonlinearity tests > 0.05).

## Graphical abstract

In 936 atrial fibrillation patients, 190 (20.3%) presented high neutrophil‐to‐lymphocyte ratio (NLR), which was independently associated with increased risks for cardiovascular death, all‐cause mortality, and the composite of major bleeding, ischemic stroke/transient ischemic attack and death over a 2‐year follow‐up. A linear relationship was observed between NLR and risk levels.

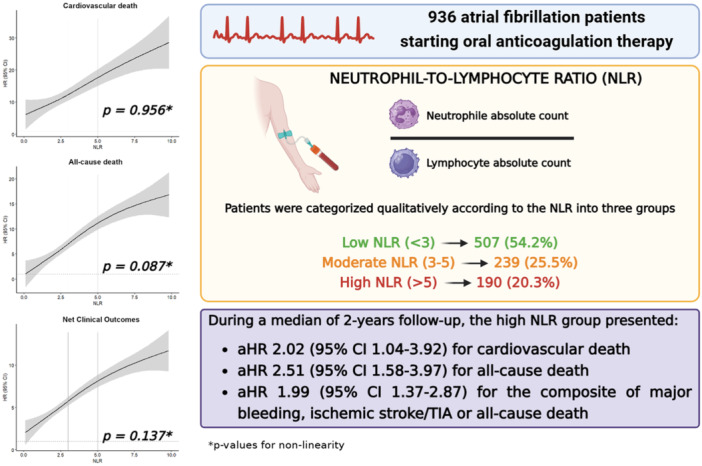

**Abbreviations**
AF: atrial fibrillation
aHR: adjusted hazard ratio
CHA_2_DS_2_‐VASc: congestive heart failure, hypertension, age ≥ 75 [doubled], diabetes mellitus, prior stroke or transient ischemic attack [doubled], vascular disease, age 65–74, female
CI: confidence interval
COPD/OSA: chronic obstructive pulmonary disease/obstructive sleep apnea
HAS‐BLED: hypertension, abnormal renal/liver function, stroke, bleeding history or predisposition, labile international normalized ratio, elderly, drugs/alcohol concomitantly
IL‐17: interleukin‐17
IL‐6: interleukin‐6
IQR: interquartile range
IRR: incidence rate ratio
MAFP‐II: Murcia AF Project II
NCO: net clinical outcomes
NLR: neutrophil‐to‐lymphocyte ratio
OAC: oral anticoagulation
TIA: transient ischemic attack
TNF‐α: tumor necrosis factor‐alpha
VKA: vitamin K antagonist

## 1 Introduction

Atrial fibrillation (AF) is the most prevalent arrhythmia, affecting approximately 1%–2% of the general population and up to 15% of individuals over 80 years old [1]. Multiple risk factors, including hypertension, valvular heart disease, heart failure, ischemic heart disease, and sleep apnea, are associated with chronic inflammation and elevated levels of inflammatory cytokines [2, 3], thereby inducing structural remodeling and electrophysiological changes in the atria [4, 5]. In patients with AF, this inflammatory state heightens the risk of thrombosis and arterial embolic events [6, 7].

Certain biomarkers have been identified as valuable predictors of adverse events in AF patients [8, 9, 10]. Given the pivotal role of inflammation, the neutrophil‐to‐lymphocyte ratio (NLR) which is the ratio between neutrophil and lymphocyte counts measured in peripheral blood, emerges as a simple and inexpensive inflammatory biomarker derived from absolute blood cell counts [11] and has been associated with adverse events in cardiovascular diseases [12, 13, 14]. Although the evidence is somewhat limited, a high NLR also appears to be associated with poor outcomes in patients with AF receiving oral anticoagulation (OAC) therapy [15].

Herein, we aimed to investigate the role of NLR as a predictive inflammatory biomarker for adverse events in a contemporary real‐world prospective cohort of AF patients.

## 2 Methods

The present study was performed within the Murcia AF Project II (MAFP‐II), which has been described in detail elsewhere [16]. In brief, this was a prospective observational cohort study including outpatients recently diagnosed with any type of AF who were treatment‐naïve for OAC and initiated vitamin K antagonist (VKA) therapy at our anticoagulation clinic from July 1, 2016, to June 30, 2018. Only adult patients (≥ 18 years old) were eligible for inclusion. Exclusion criteria encompassed patients with prosthetic heart valves, rheumatic mitral valves, or other severe valvular disease, as well as those with any concurrent inflammatory or infectious condition. No additional exclusion criteria were applied.

At inclusion, medical history was recorded, including sociodemographic and anthropometric data, comorbidities, and concomitant therapies. Furthermore, stroke and bleeding risks were assessed at baseline using the CHA_2_DS_2_‐VASc and HAS‐BLED scores, respectively.

The study protocol was approved by the Ethics Committee from the University Hospital Morales Meseguer (reference: EST:20/16) and was conducted in accordance with the ethical standards outlined in the 1964 Declaration of Helsinki and later amendments. An informed consent was required for participation.

### 2.1 Estimation of NRL and Definition of Categories

The NLR was calculated as the ratio of neutrophil to lymphocyte absolute counts obtained from the complete blood count from a blood sample drawn immediately before the start of OAC therapy. The NLR was then categorized qualitatively into three groups: low NLR (< 3), moderate NLR (3–5), and high NLR (> 5), as previously described [17].

### 2.2 Follow‐Up and Clinical Outcomes

The follow‐up period spanned 2 years. During this time, the following primary endpoints were considered: cardiovascular death, all‐cause mortality, and the net clinical outcomes (NCO), defined as the composite of ischemic stroke/transient ischemic attack (TIA), major bleeding, and all‐cause mortality. A death will be classified as cardiac‐related when there are unequivocal signs that the death occurred by a cardiovascular cause. Ischemic stroke was defined as the sudden onset of a focal neurological deficit in a location consistent with the territory of a major cerebral artery resulting from an obstruction documented by imaging, surgery, or autopsy. TIA was defined as the rapid development of clinical signs of focal or global cerebral function disturbance, lasting < 24 h, with no apparent nonvascular cause, and confirmed as positive by cerebral imaging. Major bleeding was defined according to the 2005 International Society on Thrombosis and Hemostasis criteria [18]. All clinical outcomes were identified, confirmed, and documented by the investigators. No patients were lost to follow‐up.

### 2.3 Statistical Analysis

Quantitative variables were presented as mean ± standard deviation or as median and interquartile range (IQR) when appropriate. Categorical variables were expressed as absolute frequencies and percentages. Differences in quantitative variables between groups were assessed using the Mann–Whitney *U*‐test or Student's *t*‐test, as appropriate. The Pearson's chi‐square test was used to compare proportions.

Incidence rates with their Poisson 95% confidence intervals (CIs) for the primary endpoints during the follow‐up were calculated in the different NLR categories. Incidence rates were then compared and reported as incidence rate ratios (IRRs), using the low NLR category (i.e., < 3) as the reference group.

Cox proportional hazard regression models were conducted to determine the association of the NLR categories with the risk of suffering the primary endpoints. Forward stepwise models were employed, where a univariate significance level of 0.05 was required for a variable to enter the multivariate model (SLENTRY = 0.05), and a multivariate significance level of 0.05 was required for a variable to remain in the model (SLSTAY = 0.05). Results were expressed as an adjusted hazard ratio (aHR) with a 95% CI. Survival analyses were performed using Kaplan–Meier curves, testing the difference by the log‐rank test. Kaplan–Meier survival curves were calculated using survival v. 3.6‐4R package and represented using survminer version 0.4.9.

Sensitivity analyses of time‐to‐event for the primary endpoints were also performed by using the NLR as a quantitative variable instead of using NLR categories. The analyses were performed by restricted cubic spline plots with NLR on the *X*‐axes and HR of the primary endpoints on the *Y*‐axes. The cubic spline graphs were analyzed with the adjustment of possible confounders. Therefore, the *Y*‐axes present aHR and 95% CI. Cox regression models were calculated using the survival package and graphed using ggplot2 v. 3.5.1. For the visualization of the restricted cubic spline plots, *rcs* function of rms R package v. 6.8‐1 was used.

A *p* < 0.05 was considered statistically significant. Statistical analyses were carried out using R software (R Foundation for Statistical Computing, Vienna, Austria), SPSS v. 25.0 (IBM Corp., Armonk, NY, USA), and MedCalc v. 16.4.3 (MedCalc Software bvba, Ostend, Belgium) for Windows.

## 3 Results

We included 1050 AF patients (51.5% female, median age 77, IQR: 69–83 years), with a median CHA_2_DS_2_‐VASc of 4 (IQR: 3–5) and a median HAS‐BLED of 2 (IQR: 2–3). At baseline, NLR was available in 936 patients, with a median value of 2.79 (IQR: 1.85–4.45). Of these, 507 (54.2%) patients had low NLR (< 3), 239 (25.5%) were categorized as moderate NLR (3–5), and 190 (20.3%) patients had high NLR (> 5). Patients in the higher NLR category were older and had more comorbidities, as shown in Table 1.

**Table 1:** Baseline clinical characteristics.

	Low NLR (*N* = 569)	Moderate NLR (*N* = 268)	High NLR (*N* = 213)	*p* value
Demographics				
Age (years), median (IQR)	74 (74–75)	76 (74–77)	79 (77–70)	< 0.001
Female, *n* (%)	271 (53.5)	125 (52.3)	92 (48.4)	0.495
Comorbidities, *n* (%)				
Hypertension	415 (81.9)	201 (84.1)	172 (90.5)	0.020
Diabetes mellitus	165 (32.5)	104 (43.5)	83 (43.7)	0.002
Heart failure	94 (18.5)	66 (27.6)	66 (34.7)	< 0.001
History of stroke/TIA	62 (12.2)	40 (16.7)	41 (21.6)	0.007
Vascular disease*	97 (19.1)	61 (25.5)	45 (23.7)	0.107
Renal impairment	68 (13.4)	52 (21.8)	49 (25.8)	< 0.001
Hypercholesterolemia	285 (56.2)	136 (56.9)	123 (64.7)	0.115
COPD/OSA	99 (19.5)	54 (22.6)	54 (28.4)	0.041
History of relevant bleeding	81 (16)	41 (17.2)	35 (18.4)	0.732
Liver disease	35 (6.9)	14 (5.9)	14 (7.4)	0.804
Cancer	73 (14.4)	39 (16.3)	30 (15.8)	0.765
Smoking habit	78 (15.4)	36 (15.1)	32 (16.8)	0.864
Alcoholism	33 (6.5)	21 (8.8)	14 (7.4)	0.534
Concomitant treatment, *n* (%)				
Antiarrhythmics	59 (11.6)	22 (9.2)	23 (12.1)	0.546
ACE inhibitors	113 (22.3)	67 (28)	49 (25.8)	0.209
ARBs	227 (44.8)	92 (38.5)	88 (46.3)	0.184
Calcium channel blockers	147 (29)	76 (31.8)	57 (30)	0.737
Beta‐blockers	361 (71.2)	167 (69.9)	129 (67.9)	0.691
Diuretics	248 (48.9)	143 (59.8)	119 (62.6)	< 0.001
Antilipemic agents	264 (52.1)	121 (50.6)	112 (58.9)	0.182
Oral hypoglycemic agents	100 (19.7)	73 (30.5)	55 (28.9)	< 0.001
Insulin	36 (7.1)	24 (10)	22 (11.6)	0.127
Antiplatelet therapy	104 (20.5)	67 (28)	56 (29.5)	0.014
Aspirin alone	70 (13.8)	52 (21.8)	43 (22.6)	
Clopidogrel alone	21 (4.1)	9 (3.8)	9 (4.7)	
Dual antiplatelet therapy	11 (2.2)	5 (2.1)	4 (2.1)	

Abbreviations: ACE inhibitors angiotensin‐converting‐enzyme inhibitors; ARBs, angiotensin II receptors blockers; COPD/OSA, chronic obstructive pulmonary disease/obstructive sleep apnea; IQR, interquartile range; TIA, transient ischemic attack.

* Vascular disease includes coronary artery disease and/or peripheral artery disease.

### 3.1 Primary Endpoints and Association to NLR Category

During a median follow‐up of 2 years, 63 (6.7%) died from cardiovascular cause, 149 (15.9%) died from any cause, and 232 (24.8%) patients suffered a NCO. The proportion of all the endpoints was significantly different between NLR categories and increased across the moderate‐higher NLR (*p* = 0.002 for cardiovascular death; *p* < 0.001 for all‐cause mortality, and *p* < 0.001 for NCO). The IRRs for all‐cause mortality and NCO were higher in the moderate category compared to the low category, whereas for the three endpoints, they were higher in the high category in comparison to the low category. Detailed comparisons are shown in Table 2.

**Table 2:** Incidence rate and incidence rate ratio for the different outcomes.

	Low NLR (*N* = 507)		Moderate NLR (*N* = 239)		High NLR (*N* = 190)		Low versus Moderate		Low versus High	
	*N* (%)	Incidence rate (95% CI)	*N* (%)	Incidence rate (95% CI)	*N* (%)	Incidence rate (95% CI)	IRR (95% CI)	*p* value	IRR (95% CI)	*p* value
Cardiovascular death	23 (4.5)	2.39 (1.51–3.58)	17 (7.1)	3.95 (2.30–6.33)	23 (12.1)	7.66 (4.86–11.50)	1.66 (0.83–3.23)	0.122	3.21 (1.72–5.98)	< 0.001
All‐cause death	46 (9.1)	4.78 (3.49–6.37)	41 (17.2)	9.53 (6.84–12.93)	62 (32.6)	20.65 (15.83–26.48)	2.00 (1.28–3.11)	0.002	4.32 (2.91–6.48)	< 0.001
Net clinical outcome	87 (17.2)	9.03 (7.23–11.14)	64 (26.8)	14.88 (11.46–19.00)	81 (42.6)	26.98 (21.43–33.54)	1.65 (1.17–2.30)	0.003	2.99 (2.18–4.09)	< 0.001

Abbreviations: CI, confidence interval; IRR, incidence rate ratio; NLR, neutrophil‐to‐lymphocyte ratio; TIA, transient ischemic attack.

On Cox regression analyses adjusted for several risk factors (age, sex, hypertension, diabetes, vascular disease, heart failure, previous stroke/TIA/thromboembolism, renal disease, liver disease, dyslipidemia, previous history of major bleeding, chronic obstructive pulmonary disease/obstructive sleep apnea [COPD/OSA], cancer, smoking habit, alcoholism and time in therapeutic range), the high NLR category was independently associated with a significantly increased risk of cardiovascular death (aHR: 2.02; 95% CI: 1.04–3.92; *p* = 0.038), all‐cause mortality (aHR: 2.51; 95% CI: 1.58–3.97; *p* < 0.001), and NCO (aHR: 1.99; 95% CI: 1.37–2.87; *p* < 0.001), compared to the low NLR category.

**Figure 1:**
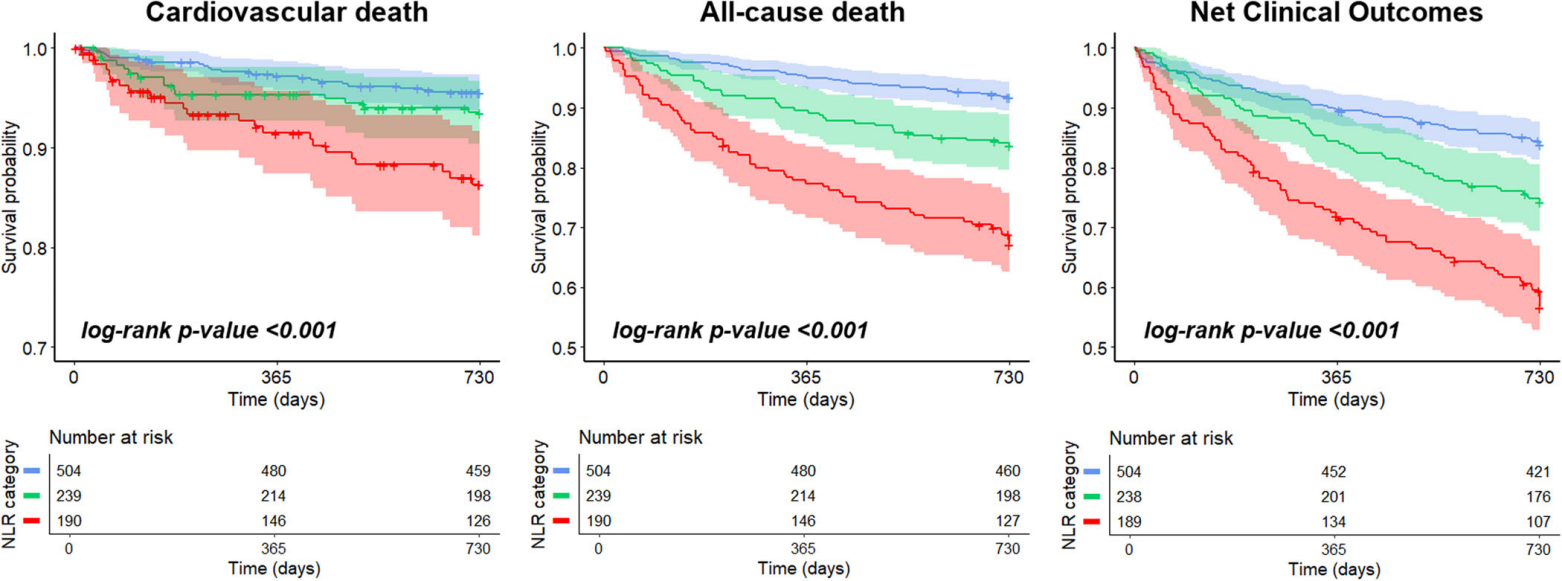
Kaplan–Meier survival curves for the primary endpoints. Blue line = low neutrophil‐to‐lymphocyte ratio (NLR) (< 3). Green line = moderate NLR (3–5). Red line = high NLR (> 5).

Kaplan–Meier analyses revealed that patients in the high NLR category exhibited lower event‐free survival rates for cardiovascular death, all‐cause mortality, and NCO than patients in the low NLR category (all log‐rank *p* < 0.001) (Figure 1).

### 3.2 Sensitivity Analysis

**Figure 2:**
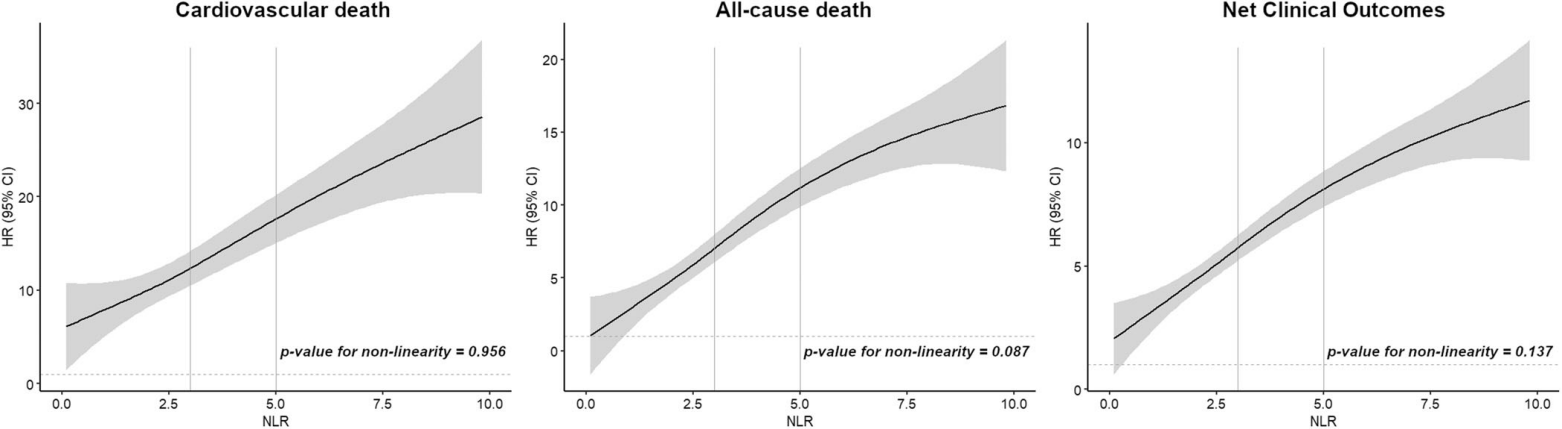
Restricted cubic spline of neutrophil‐to‐lymphocyte ratio at baseline on the *X*‐axis and adjusted hazard ratio and 95% confidence interval on the *Y*‐axis. *X*‐axes represent quantitative increases of NLR, *Y*‐axes represent adjusted hazard ratios of the primary endpoints and 95% confidence intervals. CI, confidence interval; HR, hazard ratio; NLR, neutrophil‐to‐lymphocyte ratio.

A restricted cubic spline plot was performed to demonstrate the relation of the NLR in its quantitative form with the risk of the different endpoints. In Figure 2, NLR is shown on the *X*‐axis, and the aHRs on the *Y*‐axis. All the cubic spline graphs were analyzed with the same adjustment that were used for the Cox regression analyses (i.e., age, sex, hypertension, diabetes, vascular disease, heart failure, previous stroke/TIA/thromboembolism, renal disease, liver disease, dyslipidemia, previous history of major bleeding, COPD/OSA, cancer, smoking habit, alcoholism and time in therapeutic range). As can be observed, the aHR for the tree endpoints was progressively increased as the NLR increased, showing a linear relationship between the NLR and the risk of these adverse events (*p*‐values for nonlinearity tests > 0.05) (Figure 2).

## 4 Discussion

In this prospective cohort of AF patients receiving OAC with VKAs, our main findings are as follows: (i) less than a quarter of patients presented with high NLR at AF diagnosis, and (ii) those with high NLR showed increased risks of adverse events, and (iii) the risk of worse clinical outcomes increased as the NLR increased, suggesting its potential as a biomarker for predicting all‐cause and cardiovascular mortality, as well as composite adverse events.

Biomarkers enable precise assessment of normal conditions, diseases, and treatment responses, providing central information for personalized diagnosis, monitoring, and prognosis across various pathologies. Recently, NLR has emerged as a hematimetric index that is quick, easy, and automatic to obtain through a simple peripheral blood test. It has proven to be a predictive risk biomarker in several diseases, particularly those linked with inflammation, such as cancer and sepsis [19, 20]. Additionally, the role of the NLR has been investigated in cardiovascular diseases [21], highlighting its association with worse clinical outcomes. For instance, some meta‐analyses found that a high NLR is associated with increased all‐cause mortality in heart failure patients and acute coronary syndrome patients [13, 22]. This has also been reported in patients who experienced venous thromboembolism, where NLR was associated with an increased risk of mortality [23]. Additionally, in patients hospitalized for suspected thrombosis, a high NLR is an independent predictor of arterial thrombosis [24] and is significantly related to stent thrombosis in patients with ST‐segment elevation myocardial infarction [25]. Even in patients with stroke, NLR levels are higher compared with healthy individuals, and these levels are enhanced in moderate/severe stroke compared with those with minor stroke [26].

However, the underlying mechanisms explaining the relationship between NLR and AF remain incompletely understood. A plausible hypothesis is that elevated NLR correlates with excessive activation of the interleukin‐17 (IL‐17) axis in AF [27]. IL‐17 levels, mediated by interleukin‐6 (IL‐6) produced by neutrophils, are associated with increased atrial fibrosis and production of other pro‐inflammatory cytokines such as tumor necrosis factor‐alpha (TNF‐α) [28]. NLR has been shown to be correlated with the left atrial volume index, which suggests an association on atrial remodeling [29]. These are potential reasons by which NLR is associated with an increased risk of new onset AF [30].

Regarding the potential application of NLR as a biomarker role in AF, some studies have highlighted its utility in assessing stroke risk [31]. Indeed, increased NLR elevates the risk of left atrial thrombosis in AF patients [32, 33]. However, evidence linking NLR to other clinical outcomes in AF patients is less evident. Wu and colleagues aimed to evaluate the impact of the NLR on long‐term outcomes (median follow‐up of 3.32 years) in AF patients. They showed that the highest NLR quartile was independently associated with a higher risk of all‐cause mortality (HR: 1.77; 95% CI: 1.19–2.65) and adverse cardiovascular events (HR: 1.66; 95% CI: 1.18–2.33) [34], consistent with our findings. In a subanalysis of the ENGAGE AF‐TIMI 48 trial, elevated NLR increased the risk of cardiovascular mortality (HR: 1.93; 95% CI: 1.74–2.13) and all‐cause mortality (HR: 2.00; 95% CI: 1.83–2.18), among others [15].

In summary, our study confirms previous observations regarding the usefulness of NLR in predicting worse prognosis in AF patients. Therefore, NLR emerges as a simple, inexpensive and readily available hematological parameter integrating innate and adaptive immune systems, serving as a biomarker associated with inflammation. Its significance is underscored by observations in high‐inflammatory conditions like SARS‐CoV‐2 disease, where normalization of NLR following anti‐cytokine therapy predicts treatment response and correlates with reduced major adverse cardiovascular events [35]. Considering the association we found between elevated NLR and adverse outcomes in AF patients, its integration into clinical practice could be highly beneficial. Current risk stratification tools do not directly account for inflammatory status, which may be a crucial determinant of cardiovascular risk. Our insights warrant further exploration of NLR not only as a risk prediction marker but also as an indicator of treatment response in inflammation‐related pathologies. Thus, clinicians could incorporate NLR into routine evaluations, especially for patients at intermediate risk of stroke or cardiovascular events, to personalize treatment strategies. For example, AF patients with high NLR might benefit from more aggressive risk factor management, closer follow‐up, or earlier intervention with anti‐inflammatory or anticoagulation therapies. Although many biomarkers are expensive and lack of practicality and simplicity, as well as not always available in all centers, the NLR is easily and automatically calculable based on a routine blood test available worldwide; hence, it could be introduced in routine clinical practice and aid to the management of patients with AF.

*Could NLR also predict long‐term outcomes?* The association between high NLR and long‐term, rather than short‐term, adverse outcomes may be explained by its role in chronic inflammation and atrial remodeling. There are several mechanisms that could underlie this connection. For example, elevated NLR reflects an imbalance between neutrophil‐driven inflammation and lymphocyte‐mediated immune regulation. This imbalance may contribute to persistent inflammation, atrial fibrosis, and structural remodeling, which predispose patients to recurrent AF and embolic events over time. On the other hand, neutrophil activation is also linked to increased IL‐6 and TNF‐α levels, which enhance endothelial dysfunction and promote a prothrombotic state, thereby explaining the association between high NLR and increased risk of stroke and systemic embolism [36, 37]. Finally, persistent inflammation in AF patients with high NLR could accelerate the progression of cardiovascular disease, contributing to higher mortality rates in the long term.

### 4.1 Limitations

There are several limitations related to this study. The primary limitation is its observational nature, along with a predominantly White population and single‐center design. Additionally, patient selection could be a potential limitation since only those who initiated VKA therapy were included, as this was the only OAC authorized for OAC‐naïve patients.

Furthermore, the determination of biological parameters was limited to a specific point in time (baseline), and neutrophil and lymphocyte counts are nonspecific parameters that can be affected by concurrent conditions such as infections, inflammation, and medications. Therefore, the confounding effect of concurrent inflammatory conditions cannot be completely excluded. Serial NLR measurements could help assess disease progression and guide treatment adjustments, particularly in patients with fluctuating inflammatory markers.

Lastly, we used a prespecified cut‐off for NLR, but no fixed and completely standardized cut‐off values were available. Other cut‐off values or approaches could lead to different results, and further research is needed to elucidate this issue.

## 5 Conclusions

In this prospective cohort study of AF patients starting VKA therapy, one in five patients had a high NLR at baseline. These patients had significantly increased risks of adverse clinical outcomes, including cardiovascular death, all‐cause mortality, and a composite outcome of major bleeding, ischemic stroke/TIA, and all‐cause mortality. Higher NLR at AF diagnosis demonstrated independent prognostic value beyond traditional risk.

## Author Contributions

Eva Soler‐Espejo, José Miguel Rivera‐Caravaca, Raquel López‐Gálvez, María Sánchez‐Villalobos, and María Pilar Ramos‐Bratos performed statistical analyses and drafted the manuscript. Eva Soler‐Espejo, José Miguel Rivera‐Caravaca, María Asunción Esteve‐Pastor, and Vanessa Roldán contributed to data collection. Gregory Y. H. Lip, Francisco Marín, and Vanessa Roldán conceived and supervised the study and critically revised the manuscript. All authors read and approved the final version of the manuscript.

## Conflicts of Interest

Gregory Y. H. Lip is a consultant and speaker for BMS/Pfizer, Boehringer Ingelheim, Daiichi‐Sankyo, Anthos. No fees are received personally. He is a National Institute for Health and Care Research (NIHR) Senior Investigator and co‐PI of the AFFIRMO project on multimorbidity in AF (grant agreement No 899871), TARGET project on digital twins for personalized management of atrial fibrillation and stroke (grant agreement No 101136244) and ARISTOTELES project on artificial intelligence for the management of chronic long term conditions (grant agreement No 101080189), which are all funded by the EU's Horizon Europe Research & Innovation program. José Miguel Rivera‐Caravaca is a consultant for Idorsia Pharmaceuticals Ltd. Francisco Marín is a consultant and speaker for Boehringer‐Ingelheim and BMS/Pfizer. The other authors declare no conflicts of interest.

## Data Availability

Derived data supporting the findings of this study are available from the corresponding author, José Miguel Rivera‐Caravaca, on request.
